# Margin Sampling and Survival Outcomes in Oral Cavity and p16-Positive Oropharyngeal Squamous Cell Carcinoma

**DOI:** 10.1177/2473974X221101024

**Published:** 2022-09-21

**Authors:** Colin MacKay, Brooke Turner, Martin Bullock, S. Mark Taylor, Jonathan Trites, Martin Corsten, Laurette Geldenhuys, Matthew H. Rigby

**Affiliations:** 1 Division of Otolaryngology–Head and Neck Surgery, Department of Surgery, Queen Elizabeth II Health Science Centre and Dalhousie University, Halifax, Canada; 2 Division of Anatomical Pathology, Department of Pathology, Queen Elizabeth II Health Science Centre and Dalhousie University, Halifax, Canada

**Keywords:** specimen oriented, survival outcomes, oral cavity, oropharynx, squamous cell carcinoma

## Abstract

**Objective:**

To compare the association of margin sampling technique on survival outcomes in surgically treated cT1-2 oral cavity and oropharyngeal squamous cell carcinoma.

**Study Design:**

A prospective longitudinal cohort study.

**Setting:**

Tertiary care academic teaching hospital in Halifax, Nova Scotia.

**Methods:**

All cases of surgically treated cT1-2 oral cavity and oropharyngeal cancer undergoing specimen-oriented margin analysis between January 1, 2017, and December 31, 2018 were analyzed. The specimen-oriented cohort was compared with a cohort of patients from January 1, 2009, to December 31, 2014, where a defect-oriented margin sampling protocol was used. Kaplan-Meier survival curves were used to estimate 2-year overall survival, disease-specific survival, local control, and recurrence-free survival rates in oral cavity and p16-positive oropharyngeal squamous cell carcinoma. Cox proportional hazards models were used to assess the effect of margin sampling method on disease-specific survival and local control.

**Results:**

There was no significant association between margin sampling technique and 2-year survival outcomes for surgically treated cT1-2 oral cavity and oropharyngeal squamous cell carcinoma. In the multivariate Cox proportional hazard model, the hazard ratio (HR) of specimen-oriented sampling was not significantly different for disease-specific survival (HR, 1.32; 95% CI, 0.3032-5.727; *P* = .713) or local control (HR, 0.4087; 95% CI, 0.0795-2.099; *P* = .284).

**Conclusion:**

Intraoperative margin sampling method was not associated with a significant change in 2-year survival outcomes. Despite no effect on survival outcomes, implementation of a specimen-oriented sampling method has potential for cost avoidance by decreasing the number of re-resections for positive or close margins.

Failure to eradicate disease at the primary site is well established as a major cause of mortality in head and neck cancer.^1^ While this still appears true, the landscape of oral cavity and oropharyngeal cancer in North America has undergone a notable shift in recent decades. Recent epidemiology suggests that patients are being diagnosed younger, with increasing rates among women.^2-4^ Alcohol use and smoking remain strong risks factors for head and neck cancer development; fortunately, incidence of new smokers is decreasing.^5-7^ Despite declining rates of head and neck cancers overall,^2^ rates of oropharyngeal cancer are increasing, and existing cases are predominantly driven by higher rates of human papilloma virus (HPV) infections,^1,2,5-11^ particularly high-risk subtypes HPV 16 and 18.^6,7,12,13^ HPV-positive oropharyngeal carcinoma accounts for at least 62% to 80% of all cases of oropharyngeal cancer.^2,6,14-16^ Positive p16 status has resulted in better treatment outcomes, as it appears to be more sensitive to radiation and chemotherapy.^2,14^ In addition to HPV status, several other important prognostic factors have been identified in the literature. These include the presence of cervical lymph nodes, perineural invasion, lymphovascular invasion, and extracapsular spread.^17-21^

Until recently, margin status has historically been considered the most important prognostic factor in head and neck cancer.^9,17,22-24^ Discrepancies within the literature regarding the most appropriate definition of a negative or clear margin render comparisons between margin status and survival outcomes challenging. The most widely accepted definition of a negative or clear margin is ≥5 mm from the dissection edge, while a close surgical margin is <5 mm.^18,21,22,25-28^ In contrast, some surgeons and pathologists define *close* surgical margins as <2 or <3 mm,^17,23,29^ and some go as far as to distinguish *close* (1-5 mm) and *very close* (<1 mm).^25^

Current literature highlights higher rates of local recurrence and decreased overall survival (OS) among patients in whom margins were close or positive.^9,17,23,30,31^ An analysis of 277 patients with oral cavity squamous cell carcinoma (OCSCC) found that each additional millimeter of clear margin conferred an 8% decrease in risk of death at 5 years (hazard ratio [HR], 0.92; *P* = .021).^30^ The use of frozen sections as an intraoperative margin assessment tool provides the surgeon influence of this prognostic factor by allowing re-excision intraoperatively of margins deemed close or involved.^17,32^ In a retrospective nonrandomized clinical trial, Varvares et al assessed the effectiveness of surgical margins in achieving local control (LC) in OCSCC and oropharyngeal squamous cell carcinoma (OPSCC). They identified a 3.4% local recurrence rate in patients with ≥5 mm negative margins, a 26.4% rate in patients with margins <5 mm, and a 28.6% rate in patients with initially positive margins re-resected to negative.^9^ However, the authors did not stratify survival outcomes by sampling technique, thus complicating comparisons between margin status and survival outcomes.^9^ Further confounding the landscape of literature on survival outcomes in OCSCC and OPSCC, a small subsection of literature has reported that margin status is not a significant predictor of local recurrence.^18,29^

Surveys of head and neck cancer surgeons and pathologists have estimated that >76% of surgical margin specimens sent to pathology are defect-oriented (DO).^26,33^ Several potential pitfalls of DO sampling in head and neck oncology have been reported, including a potential decrease in effectiveness with more advanced tumor stage, as well as errors in sampling and interpretation.^22,24,34,35^ Studies in the last decade have begun to compare specimen-oriented (SO) margin sampling method with the more historically used DO sampling method.^9,22,23,36,37^ In contrast to a DO sampling protocol, which samples margins from the tumor bed, an SO method involves margin sampling from the tumor specimen. Effective SO sampling requires clear, direct communication intraoperatively between pathologist and surgeon to identify margins of clinical concern, as well as a pathologist experienced in properly inking and sampling head and neck margins. Concerning margins are then inked and sampled, typically perpendicular to the tumor-margin interface.^37^ SO sampling allows for adequate assessment of actual distance of tumor cells to closest surrounding margin, as compared with DO sampling, which determines only the presence or absence of tumor cells within the tumor bed margin.^22^

Few studies in recent years have compared survival outcomes in oral cavity and oropharyngeal cancer based on margin sampling technique.^9,23,36,37^ In an assessment of T1-2N0 OCSCC, Maxwell et al found a statistically higher 3-year local recurrence-free survival (RFS) rate in patients in which margins were sampled from the specimen, as compared with tumor bed sampling (*P* = .03).^36^ In comparing positive vs negative final pathology status among 126 glossectomy specimens, Chang et al^23^ found that glossectomy margin sampling status correlated with local recurrence (*P* = .04) but that the status of tumor bed margins did not (*P* > .05).

Following the implementation of an SO sampling method, our center achieved a statistically significant decrease in the rate of positive final margins.^37^ Only a single patient out of 111 (0.9%) had a positive final pathology margin, as compared with 12.9% of patients on retrospective analysis in whom a DO protocol was used, thus supporting a higher efficacy of SO margin sampling in reducing margin positivity rate.^37^ Building on the implementation study, the objective of the following analysis is to assess the effect of margin sampling protocols on survival outcomes and recurrence in patients with T1-2 OCSCC and OPSCC.

## Methods

A prospective analysis was performed of all cases of clinical T1-2 OCSCC and OPSCC at our site, per the seventh edition of the American Joint Committee on Cancer. Cases were diagnosed and treated between January 1, 2017, and December 31, 2018, and compared with the retrospective DO cohort between January 1, 2009, and December 31, 2014, previously analyzed by Horwich et al.^37^ An SO frozen section sampling protocol was introduced at our institution on January 1, 2017, and has since become our standard operating procedure. Oropharyngeal tumors were excised by transoral laser microsurgery or transorally with monopolar cautery, depending on surgeon preference. Patients who were treated with primary radiation, patients not treated with curative intent, patients with a pathologically unidentified primary per our center’s transoral laser microsurgery protocol,^38,39^ and patients with recurrent squamous cell carcinoma were excluded from analysis. The role of adjuvant therapy was decided by a multidisciplinary tumor board and based on TNM staging, final margin status, presence of adverse pathologic features, and additional patient factors such as age and comorbidities. This decision was independent of intraoperative margin sampling method. Kaplan-Meier survival curves were used to estimate OS, disease-specific survival (DSS), LC, and RFS at 2 years after surgery. Survival outcomes were stratified by oral cavity and oropharynx. Oropharyngeal cases were further stratified by HPV p16 status given notable differences in survival outcomes. Multivariable Cox proportional hazards models were used to assess the independent effect of margin sampling method on DSS and LC. Ethics approval for the study was obtained from the Nova Scotia Health Authority Research Ethics Board (ROMEO 1020700).

## Results

Overall 153 patients with T1-2 OCSCC and OPSCC who met criteria were included in the study: 83 patients who underwent DO margin sampling and 70 patients who underwent SO sampling. There were no statistical differences in age, sex, type of adjuvant therapy, T stage, or nodal stage between the DO and SO margin sampling methods in patients with OCSCC (**Table 1**). The same was true for patients with OPSCC, apart from clinical T stage (*P* < .001; **Table 2**). There were no significant differences in overall (*P* = .115) and local (*P* = .621) recurrence rates in patients with OCSCC; 9 patients in the DO group had local or locoregional recurrence, as compared with 2 in the SO group. However, 4 patients in the oral cavity SO group had regional recurrence, as opposed to zero in the DO group.

**Table 1.** Demographics: Patients With Oral Cavity Squamous Cell Carcinoma.^a^

	DO (n = 66)	SO (n = 21)	Total (n = 87)	*P* value
Age at treatment, y				.351
Mean (SD)	63.8 (11.9)	61.0 (11.3)	63.1 (11.7)	
Range	38.0-88.0	37.0-79.0	37.0-88.0	
Sex				.438
Female	25 (37.9)	6 (28.6)	31 (35.6)	
Male	41 (62.1)	15 (71.4)	56 (64.4)	
Adjuvant therapy				.544
None	29 (43.9)	11 (52.4)	40 (46.0)	
Radiation	24 (36.4)	6 (28.6)	30 (34.5)	
Chemoradiation	2 (3.0)	2 (9.5)	4 (4.6)	
Declined/not received	3 (4.5)	1 (4.8)	4 (4.6)	
Received not otherwise specified^b^	2 (3.0)	1 (4.8)	3 (3.4)	
Reresection	6 (9.1)	0 (0.0)	6 (6.9)	
cT				.801
T1	24 (36.4)	7 (33.3)	31 (35.6)	
T2	42 (63.6)	14 (66.7)	56 (64.4)	
cN				.391
N0	48 (72.7)	16 (76.2)	64 (73.6)	
N1	4 (6.1)	3 (14.3)	7 (8.0)	
N2a	4 (6.1)	0 (0.0)	4 (4.6)	
N2b	10 (15.2)	2 (9.5)	12 (13.8)	
Recurrence				.115
No	57 (86.4)	15 (71.4)	72 (82.8)	
Yes	9 (13.6)	6 (28.6)	15 (17.2)	
Local recurrence				.621
No	57 (86.4)	19 (90.5)	76 (87.4)	
Yes	9 (13.6)	2 (9.5)	11 (12.6)	
Recurrence type				
None	57 (86.4)	15 (71.4)	72 (82.8)	
Local	3 (4.5)	1 (4.8)	4 (4.6)	
Locoregional	6 (9.1)	1 (4.8)	7 (8.0)	
Regional	0 (0.0)	4 (19.0)	4 (4.6)	
Metastatic	0 (0.0)	0 (0.0)	0 (0.0)	

Abbreviations: DO, defect-oriented; SO, specimen-oriented.

a Values are presented as No. (%) unless noted otherwise. Clinical staging based on American Joint Committee on Cancer, seventh edition.

b Unable to obtain due to lapse of research ethics board application for retrospective DO cohort.

**Table 2.** Demographics: Patients With p16-Positive Oropharyngeal Squamous Cell Carcinoma.^a^

	DO (n = 17)	SO (n = 49)	Total (n = 90)	*P* value
Age at treatment, y				.075
Mean (SD)	58.6 (8.0)	59.6 (7.9)	60.9 (8.6)	
Range	43.0-74.0	44.0-80.0	42.0-80.0	
Sex				.150
Female	2 (11.8)	10 (20.4)	20 (22.2)	
Male	15 (88.2)	39 (79.6)	70 (77.8)	
Adjuvant therapy				.247
None	2 (11.8)	11 (22.4)	22 (24.4)	
Radiation	11 (64.7)	24 (49.0)	44 (48.9)	
Chemoradiation	1 (5.9)	11 (22.4)	16 (17.8)	
Declined/not received	0 (0.0)	3 (6.1)	4 (4.4)	
Received not otherwise specified^b^	2 (11.8)	0 (0.0)	2 (2.2)	
Reresection	1 (5.9)	0 (0.0)	2 (2.2)	
cT				<.001
T1	11 (64.7)	5 (10.2)	26 (28.9)	
T2	6 (35.3)	17 (34.7)	37 (41.1)	
Tx	0 (0.0)	27 (55.1)	27 (30.0)	
cN				.053
N0	2 (11.8)	3 (6.1)	14 (15.6)	
N1	4 (23.5)	22 (44.9)	28 (31.1)	
N2	0 (0.0)	2 (4.1)	2 (2.2)	
N2a	2 (11.8)	6 (12.2)	13 (14.4)	
N2b	6 (35.3)	12 (24.5)	23 (25.6)	
N2c	1 (5.9)	1 (2.0)	3 (3.3)	
N3	2 (11.8)	3 (6.1)	7 (7.8)	
Recurrence				.041
No	14 (82.4)	45 (91.8)	79 (87.8)	
Yes	3 (17.6	4 (8.2)	11 (12.2)	
Local recurrence				.432
No	15 (88.2)	48 (98.0)	85 (94.4)	
Yes	2 (11.8)	1 (2.0)	5 (5.6)	
Recurrence type				.002
None	14 (82.4)	45 (91.8)	79 (87.8)	
Local	1 (5.9)	0 (0.0)	3 (3.3)	
Locoregional	1 (5.9)	1 (2.0)	2 (2.2)	
Regional	0 (0.0)	2 (4.1)	2 (2.2)	
Metastatic	1 (5.9)	1 (2.0)	4 (3.3)	

Abbreviations: DO, defect-oriented; SO, specimen-oriented.

a Values are presented as No. (%) unless noted otherwise. Clinical staging based on American Joint Committee on Cancer, seventh edition.

b Unable to obtain due to lapse of research ethics board application for retrospective DO cohort.

### Oral Cavity Squamous Cell Carcinoma

**Figure 1.**
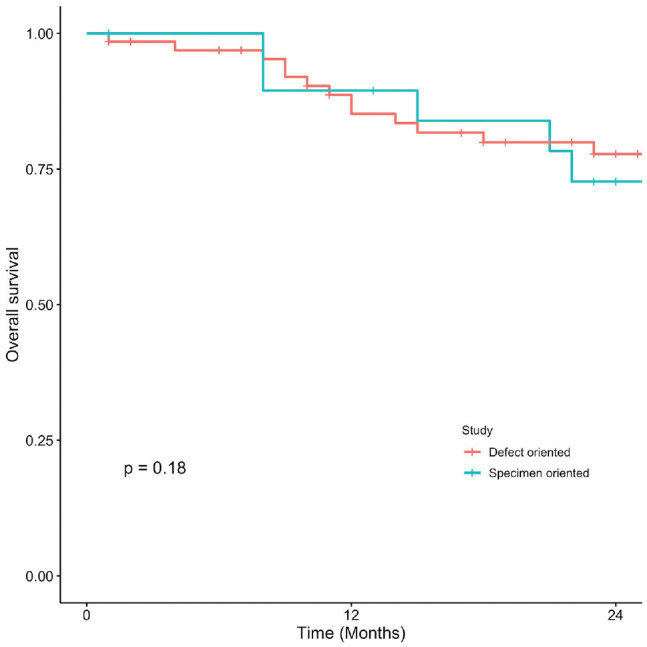
Oral cavity squamous cell carcinoma: 2-year overall survival.

**Figure 2.**
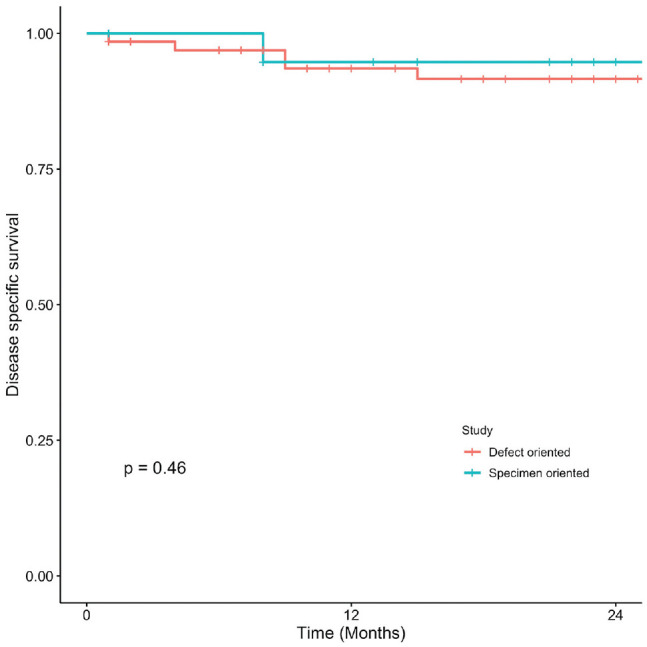
Oral cavity squamous cell carcinoma: 2-year disease-specific survival.

**Figure 3.**
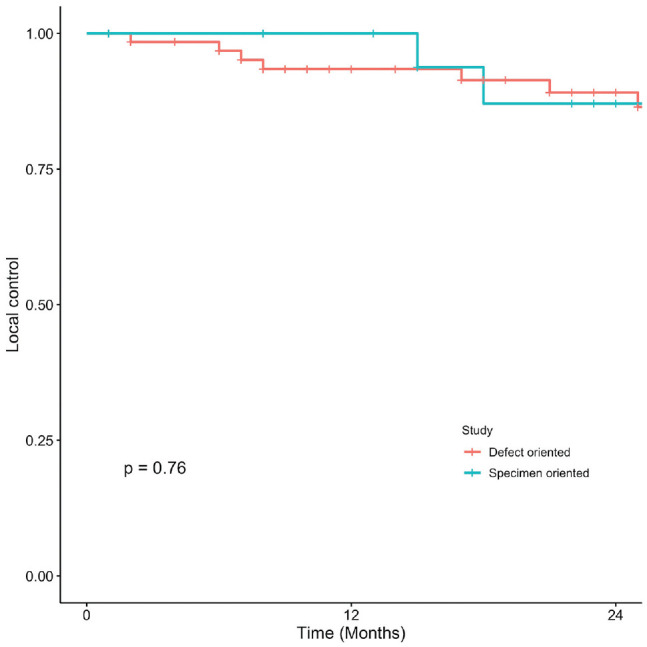
Oral cavity squamous cell carcinoma: 2-year local control.

**Figure 4.**
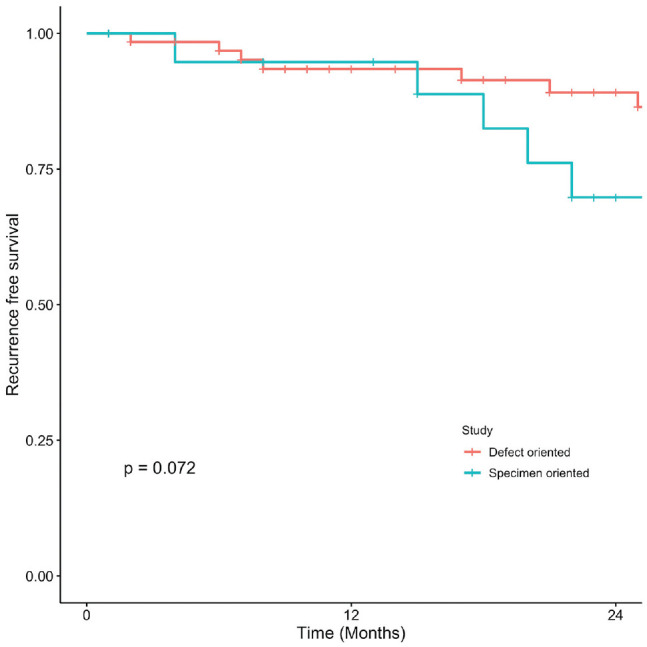
Oral cavity squamous cell carcinoma: 2-year recurrence-free survival.

Kaplan-Meier curves present OS, DSS, LC, and RFS for OCSCC (**Figures 1**-**4**, respectively). There was no statistically significant difference in OS between margin sampling methods in OCSCC. Two-year OS in patients who underwent DO sampling was 77.8% (SE, 7.1%; 95% CI, 67.7%-89.3%; n = 66), whereas for patients who underwent SO sampling it was 72.7% (SE, 14.4%; 95% CI, 54.9%-96.3%; n = 21; *P* = .18; **Figure 1**). The same was true for 2-year DSS in oral cavity tumors (*P* = .46). Two-year DSS in the DO study arm was 91.6% (SE, 3.9%; 95% CI, 84.8%-99%; n = 66) as compared with 94.7% (SE, 5.4%; 95% CI, 85.2%-100%; n = 21) in the SO arm (*P* = .46; **Figure 2**). Two-year LC for patients in the DO study arm was 89.1% (SE, 4.8%; 95% CI, 81.1%-97.9%; n = 66) vs 87.1% (SE, 9.8%; 95% CI, 71.8%-100%; n = 21) for patients in the SO study arm (*P* = .76; **Figure 3**). Last, 2-year RFS in the DO and SO arms was 89.1% (SE, 4.8%; 95% CI, 81.1%-97.9%; n = 66) and 69.8% (SE, 16.3%; 95% CI, 50.7%-96.1%; n = 21), respectively (*P* = .072; **Figure 4**).

### P16-Positive OPSCC

**Figure 5.**
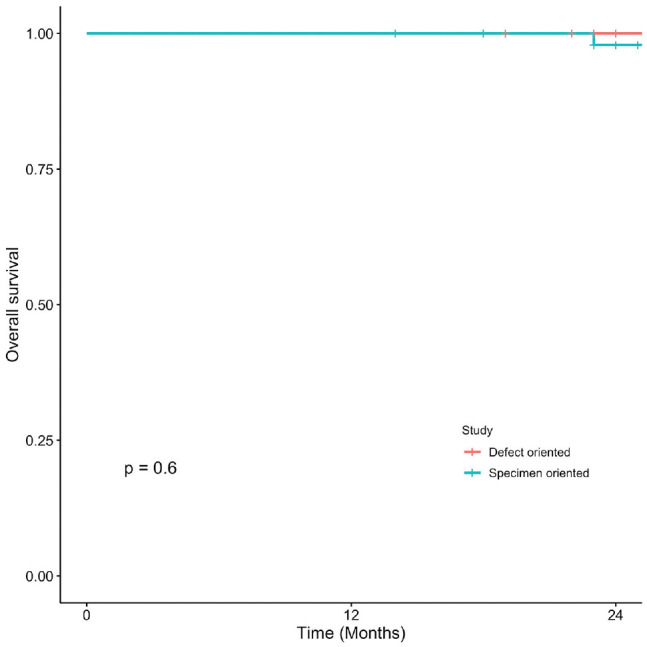
p16-Positive oropharyngeal squamous cell carcinoma: 2-year overall survival.

**Figure 6.**
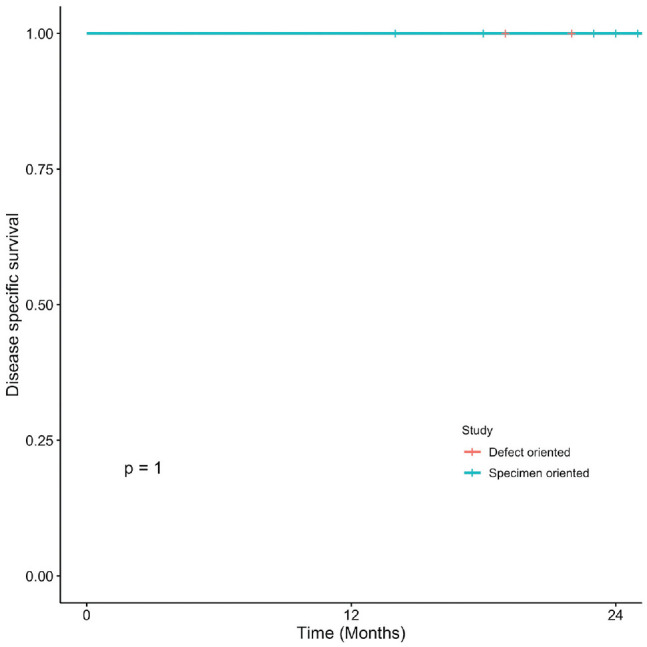
p16-Positive oropharyngeal squamous cell carcinoma: 2-year disease-specific survival.

**Figure 7.**
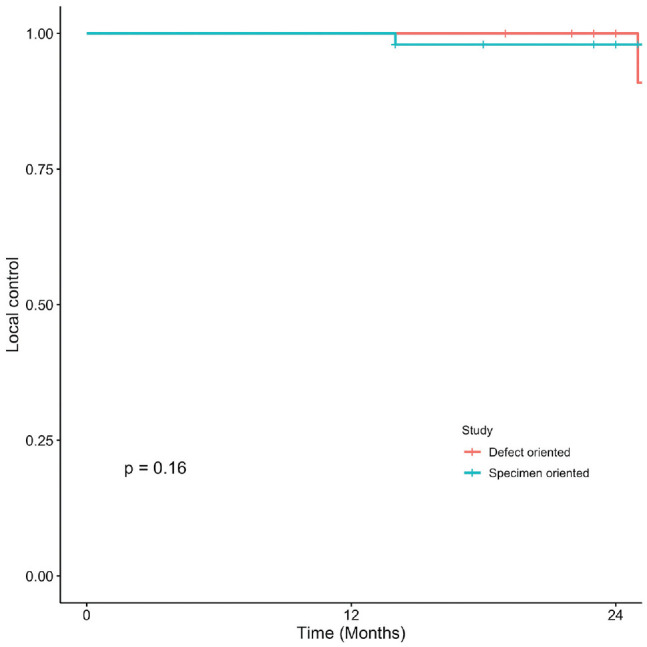
p16-Positive oropharyngeal squamous cell carcinoma: 2-year local control.

**Figure 8.**
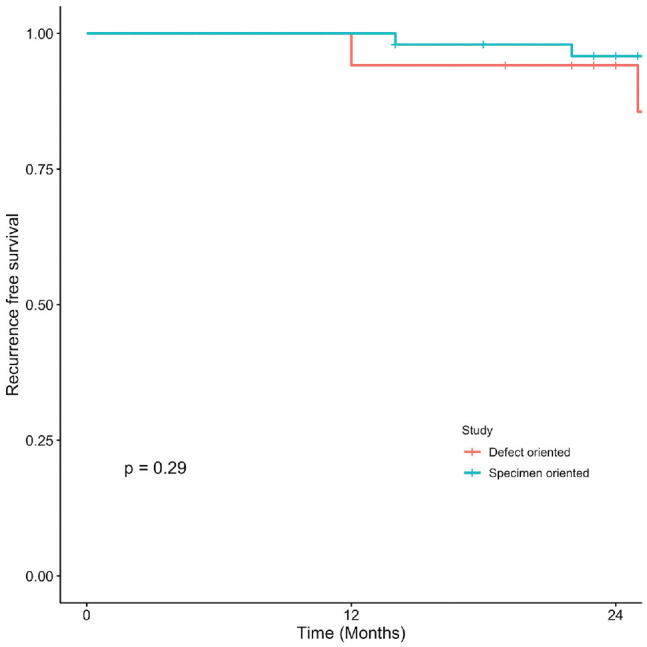
p16-Positive oropharyngeal squamous cell carcinoma: 2-year recurrence-free survival.

Survival outcomes for p16-positive OPSCC in our study population are presented in **Figures 5** to **8**. Two-year OS for patients in the DO study arm was 100% (SE, 0%; 95% CI, 100%-100%; n = 17) while for patients in the SO study arm, it was 97% (SE, 2.2%; 95% CI, 93.8%-100%; n = 49; *P* = .6; **Figure 5**). Two-year DSS for both study arms was 100% with a standard error of 0% (*P* > .99; **Figure 6**). Two-year LC rates for DO and SO sampling were 100% (SE, 0%; 95% CI, 100%-100%; n = 17) and 98% (SE, 2.1%; 95% CI, 94.1%-100%; n = 49), respectively (*P* = .16; **Figure 7**). Last, 2-year RFS in the DO and SO arms was 94.1% (SE, 6.1%; 95% CI, 83.6%-100%; n = 17) and 95.8% (SE, 3%; 95% CI, 90.2%-100%; n = 49; *P* = .29; **Figure 8**).

### p16-Negative OPSCC

Due to a low number of patients with p16-negative OPSCC in both study arms (n = 3 in each), 95% CIs were notably large at 6.7% to 100% for OS and RFS in the SO cohort. Kaplan-Meier curves were not presented as such large standard errors are of minimal prognostic value.

### Cox Proportional Hazards

Cox proportional hazards models were used to determine the impact of SO sampling on DSS and LC. The model examining the effect of margin sampling method on DSS and LC controlled for age at treatment, cT stage, cN stage, and subsite/p16 status. In the final model, SO margin sampling did not significantly increase the hazard of DSS (HR, 1.32; SE, 0.7497; 95% CI, 0.3032-5.727; *P* = .713) and did not significantly decrease the hazard of local recurrence (HR, 0.4087; SE, 0.8349; 95% CI, 0.0795-2.099; *P* = .284).

## Discussion

Few studies to date have aimed to assess the impact of SO margin sampling on survival outcomes in OCSCC and OPSCC. Despite a significant decrease in final margin positivity rate at our institution based on an SO sampling method,^37^ the transition to SO sampling was not associated with improved LC rates or improved 2-year survival outcomes in surgically treated patients with OCSCC and p16-positive OPSCC. The inability of SO sampling to improve survival outcomes to a measurable degree was disappointing. While this may simply represent the study being underpowered to detect a difference in survival outcomes, the current analysis led us to consider the interplay between prognostic factors outside the surgeons’ control and tumor at the dissection edge, also referred to as tumor cut-through. This has been reported in the literature, as Patel et al found tumor cut-through to be associated with extracapsular spread (*P* = .003).^21^ Re-excision in these cases may not completely overcome the effects of other associated negative prognostic factors and bring survival outcomes up to those expected for tumors resected with an initially negative margin. Again, this was identified by Patel et al, where the need for re-resection to achieve negative margins was associated with worse survival outcomes in patients with regional metastatic disease but not in those without regional metastases.

Our institution’s DO negative margin rate^37^ of 87% was higher than the DO and SO negative margin rates of Amit et al (55% and 84%).^22^ In addition, DSS, LC, and RFS outcomes were already high in our DO population. Power calculations demonstrated that our study was underpowered. These were estimates only, as the calculations required median survival rates and these were unavailable for many of our outcomes with the observed survival and recurrence rates. Given the underpowered study, the low number of events witnessed during follow-up (death or recurrence), and the low positive margin rate, it is unsurprising that we were unable to detect a statistically significant difference in HR for death or recurrence. The lack of improvement after the transition to an SO protocol and the decrease in our positive margin rate from 12.9% to <1% may also indicate that we may have already been achieving most or all the survival benefits of adequate resection prior to implementation. Potentially supporting this is that our institution’s DO and SO LC rates in p16-positive OPSCC are consistent with those reported by Hinni et al^40^ in their margin mapping assessment of 128 tonsil cancers. These authors reiterate the importance of close communication between surgeon and pathologist during intraoperative margin assessment.

Lymphovascular invasion and perineural invasion were not included in our analysis but are adverse pathologic features that warrant consideration for radiation therapy per the NCCN Guidelines in early-stage oral cavity and oropharyngeal cancer, even in patients with negative surgical margins.^41^ An additional pathologic factor not included in our analysis but one that has been shown to affect survival outcomes in OCSCC is worst pattern of invasion (WPOI).^19,42,43^ High-risk aggressive tumors (WPOI 4 and 5) were associated with decreased 3-year disease-free survival as compared with nonaggressive tumors (WPOI 1-3) in a study of pT1-2 node-negative oral cavity cancer (*P* = .035).^44^ Using a histopathologic risk assessment model based on perineural invasion, WPOI, and lymphocytic host response, clinicians at our institution previously determined risk of recurrence in low-, intermediate-, and high-risk pathologic categories in early-stage OCSCC.^19^ Locoregional recurrence was significantly higher in high-risk categories vs low/intermediate-risk categories (*P* < .001).^19^ After controlling for margin status and T stage, high-risk tumor categories were associated with a recurrence odds ratio of 12.4 (*P* < .001).^19^ Unfortunately we did not record these pathologic factors prospectively as part of our current study.

The American Joint Committee on Cancer released its eighth edition 1 year after the implementation of an SO margin sampling protocol at our institution. The impact of depth of invasion and extranodal extension, 2 pathologic features added to the oral cavity tumor staging, on survival outcomes in our study is unknown as they were not included in our analysis.^45,46^ The inclusion of depth of invasion in the eighth edition has the potential to upstage pathologic T stage.^47^ Additionally, the eighth edition may stage clinical TNM status higher than pathologic TNM status; this becomes more obvious in more advanced disease. Pathologic nodal status was not recorded for the retrospective DO cohort; therefore, we could only complete our comparative analysis using clinical T and N stages only (**Table 1**). As a result, the implications of using clinical vs pathologic TNM staging on our survival outcomes analysis are unknown.

Although the use of an SO protocol had no significant effect on survival outcomes, the potential health care costs avoided cannot be understated. As outlined in our previous work, use of an SO protocol would have resulted in an estimated cost avoidance of CAD $412,052.81 in the retrospective cohort, particularly due to a potential decrease in re-resections for positive margins.^37^ This breaks down to a potential cost avoidance of CAD $298,965.70 and $103,028.41 in the oral cavity and oropharynx groups, respectively. Our previous findings support those of Amit et al.^22^ In a cohort of 71 patients who underwent primary surgery for OCSCC, the authors noted that in the absence of other concerning pathologic features, SO sampling conferred a 30% reduction in escalation of therapy, in the form of re-resection and/or radiation therapy. As SO sampling does not require a significant increase in processing time and does not significantly increase hazard of death (HR, 2.33; *P* = .06),^37^ it is an appropriate alternative to the historically used DO sampling technique and confers additional pathologic information, specifically exact distance from margin to tumor cells.

## Conclusion

Despite a significant decrease in margin positivity rate^37^ and promising LC rates, SO sampling was not associated with an improvement in 2-year survival outcomes. The analysis suggests that initial re-resection guided by SO intraoperative assessment may not negate the effects of other pathologic factors that can be associated with initial tumor cut-through, such as regional disease, extranodal extension, and WPOI, which are also associated with a more aggressive disease course.^21^ Despite this, SO margin sampling has important potential for cost avoidance by reducing re-resections for positive or close margins. With the health care system currently under immense pressure and financial strain, consideration for cost avoidance for physicians is becoming even more crucial.

## Author Contributions

**Colin MacKay**, data acquisition, statistical analysis, and revision of the manuscript; **Brooke Turner**, analysis, interpretation, drafting, and revision of manuscript; **Martin Bullock**, interpretation of tumor pathology reports and assistance with revision of the manuscript; **S. Mark Taylor**, data acquisition and assistance with revision of manuscript; **Jonathan Trites**, data acquisition and assistance with revision of manuscript; **Martin Corsten**, data acquisition and assistance with revision of manuscript; **Laurette Geldenhuys**, interpretation of tumor pathology reports and assistance with revision of the manuscript; **Matthew H. Rigby**, study conceptualization, researcher supervisor, assistance with data acquisition, analysis, interpretation, preparation, and revision of the manuscript.

## Disclosures

**Competing interests:** None.

**Sponsorships:** This work was supported by the QEII Foundation Translating Research Into Care Healthcare Improvement Research Funding Program (grant 1025203, 2019).

**Funding source:** None.
